# Alcohol, tobacco and cannabis use in adolescence – Cross-sectional results of the 2017/18 HBSC study

**DOI:** 10.25646/6903

**Published:** 2020-09-16

**Authors:** Irene Moor, Kristina Winter, Katharina Rathmann, Ulrike Ravens-Sieberer, Matthias Richter

**Affiliations:** 1 Martin Luther University Halle-Wittenberg, Medical Faculty, Institute of Medical Sociology; 2 Fulda University of Applied Sciences, Department of Nursing and Health Science; 3 University Medical Center Hamburg-Eppendorf, Center for Psychosocial Medicine, Department of Child and Adolescent Psychiatry, Psychotherapy and Psychosomatics

**Keywords:** SMOKING, TOBACCO, ALCOHOL, CANNABIS, SUBSTANCE USE, CHILDREN AND ADOLESCENTS, SCHOOL, HBSC

## Abstract

Tobacco, alcohol and cannabis are psychoactive substances that is often tried for the first time during adolescence and further continued in later life. Regular tobacco and cannabis use as well as alcohol abuse are associated with serious health consequences. According to the importance of health reporting, this article describes current prevalence of adolescent substance use and the associations between psychoactive substance use and specific social determinants. Representative data for Germany from the 2017/18 Health Behaviour in School-Aged Children (HBSC) study among schoolchildren aged 11, 13 and 15 years are used. The article analyses both, the lifetime and 30-day prevalence of tobacco, alcohol and cannabis use (in the latter case, data were only available for 15-year-olds) among adolescents as well as their experiences of alcohol-related misuse (binge drinking). Tobacco and alcohol are used comparatively rarely by 11- and 13-year-olds. However, the prevalence increases significantly among 15-year-olds. In addition, cannabis use is also quite common among this age group. Schoolchildren who do not attend grammar schools are at greater risk of smoking and those with high family affluence are at a greater risk of alcohol use, this applies particularly to girls. Finally, adolescents with a migration background are less at risk of regular alcohol use or binge drinking, but face an increased risk of cannabis use (girls with one-sided migration background). The results indicate that prevention measures should start early, as the prevalence of substance use is significantly higher among older schoolchildren. Depending on the substance, different risk groups can be identified that require particular consideration when drawing up preventive measures.

## 1. Introduction

In our society, cigarettes and alcohol are ‘common drugs’ that are part of people’s everyday lives. These drugs are legal and have been widespread in our culture for centuries. In contrast, drug policy relating to cannabis remains a contentious issue and debates about it are often conducted on an emotional level [1, 2]. A focus on adolescence is particularly important in this context because people usually come into contact with psychoactive substances during this phase of life for the first time [3, 4]. Young people have to learn – consciously and subconsciously – how to handle these substances. At the same time, adolescents often want to set themselves apart from family or school norms, try out new forms of behaviour, overstep boundaries and experience risk. This leads them to adopt (or perhaps reject) numerous health-related attitudes and behavioural patterns [5]. Most forms of behaviour and habits that are ‘successfully’ adopted in adolescence continue to be practiced throughout adulthood and, therefore, determine future health [6]. Substance use poses a particular problem if it begins very early or if it’s excessive, or if it occurs in combination with other problematic forms of behaviour [7]. Excessive alcohol use is often associated with (fatal) accidents, (sexual) violence, aggression, physical and emotional problems, developmental risks, suicide (attempts), unwanted pregnancies, decline in schoolperformance, truancy and the use of other (illegal) substances [8–13]. At the same time, alcohol abuse and tobacco use are among the key risk factors of morbidity and premature mortality throughout the world [14, 15]. Further, cannabis is the most widespread (illegal) drug used by adolescents in Europe [16], and it poses serious risks to healthy development and coping with the developmental tasks associated with this life stage [17].

During the past 15 years, drastic changes have occurred to the prevalences of substance use. The results from the Health Behaviour in School-aged Children (HBSC) study and the German Health Interview and Examination Study for Children and Adolescents (KiGGS) show clear positive trends for tobacco and alcohol use [18]. The results from the Drug Affinity Study by the Federal Centre for Health Education (BZgA) demonstrate a comparable trend: the proportion of young people aged between 12 and 17 who smoke has declined sharply. In fact, the proportion of smokers decreased from 27.5% in 2001 to 6.6% in 2018. Moreover, the proportion of adolescents who have never smoked was 82.7% in 2018; the highest that it has ever been [19]. The study also found a decline in regular alcohol use and binge drinking among this age group during the past 15 years [20]. Similarly, the HBSC study also found a comparable trend for alcohol use in many Western European countries [21]. Nevertheless, as results from the BZgA illustrate, the data on cannabis use paints a different picture: between 1997 and 2004 lifetime prevalence of cannabis use among 12- to 17-year-olds increased significantly to 15%; by 2011, the prevalence had dropped to 7%. However, the prevalence has increased again since then, and it was around 10% in this age group in 2018. Boys have more experience with cannabis use than girls (12% vs. 7% in 2018) [22].

Reporting current prevelances of substance use, including stratification by certain risk groups, is important for health monitoring and for verification whether the goals of preventive strategies have been achieved [23]. The regularly generated results from the HBSC study allow to be stratified by wide-ranging social factors and to be compared the data from KiGGS and the Drug Affinity studies, despite the fact that they applied different sample designs. The results of the HBSC study indicate that substance use increases significantly among young people with increasing age. Therefore, it is important to determine which age groups particularly need to be addressed when drawing up preventive measures. Girls and boys also demonstrate different patterns of substance use [24, 25] as well as other social factors such as education level (school type), socioeconomic background and ethnic origin also appear to play a role in psychoactive substance use [18–20, 22, 24, 26]. Therefore, this article reports current cross-sectional findings on the prevalence of tobacco, alcohol and cannabis use among 11-, 13- and 15-year-olds. It also examines whether and the extent to which the use of these substances varies by sex, age, school type, family affluence and migration background.

## 2. Methodology

### 2.1 Sample design and study implementation

The HBSC study is one of the world’s largest studies of child and adolescent health. It involves the collection of data from young people aged 11, 13 and 15 years every four years. Implementation follows internationally established guidelines. The HBSC study was launched in 1982, and, since then, 50 countries have joined the HBSC network. A total of 45 countries participated in the 2017/18 cycle. Germany began participating in the study in 1994, but only its most populous state, North Rhine-Westphalia (NRW), took part. Since then, other federal states have followed, and the HBSC study has been carried out in all federal states since the 2013/14 cycle. As such, the results are now representative of Germany as a whole. The age groups under study largely correspond to the fifth, seventh and ninth grades in German schools. The study uses a stratified cluster sample, with schools constituting the first unit and school classes the second. The sample is representative for Germany, both in terms of federal state and school type. The 2017/18 survey cycle was conducted using two supplementary samples (one from Brandenburg and one from Saxony-Anhalt) in addition to a full urban sample from the town of Stuttgart. In Germany, the HBSC study was approved in advance by the education ministries of each federal state (with the exception of North Rhine-Westphalia, where the decision lay with the school administration) and, depending on state regulation, in consultation with the state’s data protection officer. Planning and coordination of the standardised survey was carried out by the German team in Halle (Saale), with recruitment undertaken decentrally at all HBSC study locations in Germany. In order to ensure that the survey was standardised in all participating schools, wide-ranging information and instructions were made available to the school and teaching staff for the day of the survey. Detailed information on the methodology applied by the German HBSC study can be found in the article by Moor et al. in this issue of the Journal of Health Monitoring.

### 2.2 Survey instruments

#### Indicators of substance use

In order to measure tobacco and alcohol use, schoolchildren were asked on how many days (if any) they had smoked cigarettes or had drunk alcohol. A distinction was made between lifetime prevalence (‘in your entire life’) and current prevalence (‘in the last 30 days’). The seven-step answer scale ranged from ‘never’ to ‘30 days or more’. The study analysed lifetime prevalence (at least once in a person’s life), current prevalence (at least once in the last 30 days) and daily smoking (during the last 30 days).

Data on binge drinking was gathered by asking school-children whether they had ever consumed so much alcohol that they had become drunk. The seven-step answer scale ranged from ‘never’ to ‘more than 10 times’ [27]. Once again, lifetime prevalence (been drunk in your entire life) and the 30-day prevalence (at least once in the last 30 days) were obtained.

Data on cannabis use were only collected from 15-year-olds. The adolescents were able to state whether they had ever used cannabis, hashish or marijuana. The seven-step answer scale ranged from ‘never’ to ‘30 days or more’. Lifetime prevalence (in your entire life) and the 30-day prevalence (at least once in the last 30 days) were also analysed in this case.

**Info box:**

**German secondary school system**

This paper includes terminology specific to the German secondary school system, whereby students can attend different schools that vary in their level of academic and/or vocational focus. In general, a Hauptschule is attended by students aged 10 to 16 and offers a basic general education, a Realschule provides a more extensive education for students aged between 10 and 16. A Gymnasium teaches students aged between 10 and 19, provides an in-depth general education and is focused on preparing students for higher education.

Gemeinschaftschulen are secondary education schools, primarily for students aged 10 to 16, where students learn together and are able to sit the same qualifications offered in the three other school types (Hauptschule, Realschule and Gymnasium).

#### Sociodemographic and socioeconomic factors

Sex, age, migration background, school type and family affluence were taken as sociodemographic and socioeconomic factors.

School type was not recorded directly on the questionnaire, but by the schools themselves when returning the survey materials. As numerous types of school exist at the federal state level (Info box), schools were categorised either as grammar school or other types of school. [28] The HBSC study has developed a means of measuring family affluence that involves asking schoolchildren questions that they can answer easily [29–32]. The Family Affluence Scale (FAS), as it is known, has been continuously adapted over the past 20 years to fit the constantly changing lives and environments of children and adolescents [32, 33]. The FAS was operationalised with the help of six items (car ownership, own (bed)room, holiday with the family, computer ownership, number of bathrooms and owning a dishwasher), and the answers were scored and added up. A relative measure was used for the analyses, which led the FAS to be divided into three categories indicating either low (the lowest 20% of the sample), medium (the mid-60% of the sample) or high (the upper 20% of the sample) family affluence [27, 32]. The HBSC study also asked questions about migration background, where the adolescents were able to state their country of birth, and that/those of their mother and father. An adolescent with one parent born outside of Germany was referred to as having a one-sided migration background. Adolescents were said to have a two-sided migration background if a) the adolescent itself was not born in Germany and at least one parent was not born in Germany or b) both parents had moved to Germany and were not born in Germany. In all other cases, the adolescents were categorised as having no migration background.

The respective operationalisation can be found in detail in Moor et al. in this issue of the Journal of Health Monitoring.

### 2.3 Statistical analysis

The descriptive analyses of tobacco, alcohol and cannabis use are differentiated by sex, age and school type (Table 1 and Table 2). All analyses for cannabis use were based on a sub-sample of 15-year-olds. In order to determine the relationship between substance use and sociodemographic and socioeconomic factors, binary-logistic models were applied once missing values from the respective variables had been excluded. Separate logistic analyses were initially carried out for model 1 that controlled for age, whereas each sociodemographic and socioeconomic variable was controlled for in model 2 (Table 3 and Table 4). The tables include odds ratios (OR) with 95% confidence intervals. The OR provided indicate the factor by which the statistical chance of a health outcome (e.g. regular tobacco or alcohol use and binge drinking) occurring in a particular group is higher than in the reference group. Since this article focuses on risk behaviour, the term ‘risk’ is used rather than ‘statistical chance’ to ensure that the results are more comparable with the literature. The respective reference category is specified in each model. With the exception of calculations of the absolute number of cases, the analyses were carried out using a weighting factor that adjusted for deviations within the sample from the population structure with regard to age, sex, school type and federal state. All analyses were carried out using IBM SPSS 25 and differentiated by sex.

## 3. Results

Information about the sample distribution in terms of sociodemographic and socioeconomic variables (age, sex, family affluence, school type and migration background) can be found in the article by Moor et al. in this issue of the Journal of Health Monitoring. Table 1 demonstrates that 14.5% of adolescents have smoked a cigarette at least once in their lives and that 6.7% have done so in the past 30 days. Only a very small proportion of adolescents (1.3%) have smoked daily, and, in general, sex-specific differences are small. About over one third of adolescents have tried alcohol at least once in their lives, with almost a quarter having drunk alcohol at least once in the past 30 days. 17.1% have reported experiences with binge drinking (lifetime prevalence), of which 7.4% of girls and 8.5% of boys have done so in the previous 30 days. Among 15-year-olds, 15.5% of girls and 22.6% of boys have used cannabis at least once in their lives, with around half as many have done so in the last 30 days.

Substance use clearly depends on age, and this applies to both sexes (Table 2). At the age of 11, 1.1% of girls have ever smoked, whereas 8.2% of 13-year-old girls and almost one third of 15-year-old girls have done so. 14.8% of 15-year-old girls currently smoke (30-day prevalence), but only a small proportion (3.3%) smokes every day. The prevalences are very similar among boys. With regard to alcohol use, prevalence increases significantly with age, although 4.5% of girls and 12.9% of boys have already tried alcohol by the age of 11. By the age of 13, almost one third of young people have drunk alcohol at least once, whereas the lifetime prevalence is over 70% among 15-year-olds. Around half of 15-year-olds surveyed stated that they had drunk alcohol in the past 30 days. Less than 2% of 11-year-olds reported having experienced binge drinking. 5.3% of 13-year-old girls and 7.8% of 15-year-old boys have been drunk at least once. These figures increase significantly with age: 40.4% of 15-year-old girls and 43.0% of boys of the same age have been drunk at least once in their lives. Of these, 18.4% of girls and 22.8% of boys were drunk at least once in the past 30 days. Differences between the sexes were also identified for cannabis use among 15-year-olds: 15.5% of girls and 22.6% of boys have used cannabis at least once in their lives, and about half of these adolescents are current cannabis users (30-day prevalence).

**Figure 1:**
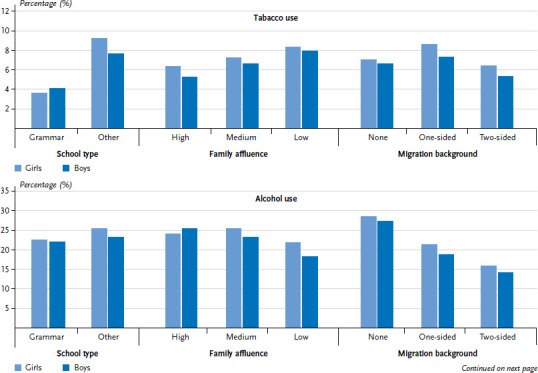

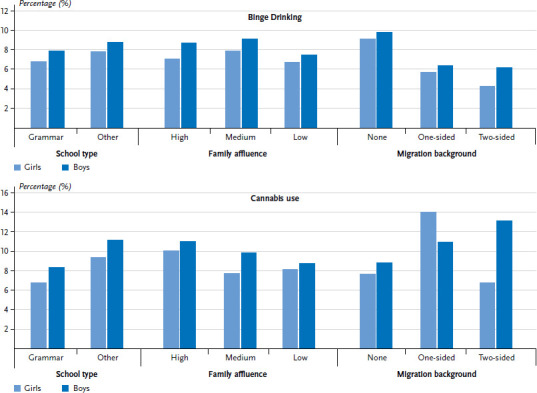
30-day prevalence of substance use by sex, school type, family affluence and migration background (n=2,306 girls, n=2,041 boys) Source: 2017/18 German HBSC study

Since this article focuses on current adolescent substance use, Figure 1 only sets out the 30-day prevalence and does so by school type, family affluence and migration background for girls and boys. Differences are identifiable with regard to tobacco use for both sexes by school type: pupils who do not attend grammar schools smoke more often than those who attend other schools. There are only slight differences by school type for alcohol and cannabis use. Regarding alcohol use, adolescents with high family affluence – and this particularly applies to boys – consume alcohol to a greater extent than those with lower family affluence. In contrast, clear differences are identifiable with regard to migration background: pupils with a migration background consume less alcohol but have more experience with cannabis compared to those without a migration background (in the case of girls, this only applies to those with a one-sided migration background).

###  

#### The associations between tobacco, alcohol and cannabis use and sociodemographic/socioeconomic variables

Logistic regression analyses (Table 3 and Table 4) were used to test the bivariate relationships described above with regard to current substance use (30-day prevalence). Age plays the greatest role in adolescent tobacco and alcohol use, regardless of other sociodemographic and socioeconomic variables (model 1 and model 2). 15-year-old girls and boys have a significantly greater risk of tobacco or alcohol use than 11- or 13-year-olds. In addition, school type plays a significant role for both sexes when it comes to tobacco (model 2). Adolescents who do not attend a grammar school are twice as likely to be current smokers as those who do. This relationship is somewhat more pronounced among girls. No differences were identified for alcohol or cannabis use by school type. With regard to family affluence, girls with low family affluence use alcohol significantly less frequently than those with high family affluence. This difference is also identifiable among boys (model 1), but not by school type or migration background (model 2). No further relationships were identified between family affluence and other substances. However, differences were found for migration background, particularly among girls. Girls with a one- or two-sided migration background have a lower risk of alcohol use or of experiencing binge drinking. The same applies for alcohol use by boys with a migration background; however, when it comes to binge drinking, only boys with a two-sided migration background did not get drunk as often as those with a one-sided or no migration background. Girls with a one-sided migration background are twice as likely to be current cannabis users as adolescents with no migration background. No similar association was identified among boys.

## 4. Discussion

###  

#### Summary of results

The results of the HBSC study show that experiences with tobacco, alcohol and cannabis are still widespread, especially among 15-year-olds. With the exception of cannabis, no significant sex-specific differences were identified for substance use. In contrast, clear age-dependant differences were found for alcohol and tobacco. Whereas the proportion of 11-year-olds who consume tobacco is rather low, both lifetime prevalence and current use (in the past 30 days) increase significantly among 13-year-olds and particularly among 15-year-olds. Almost a third of girls and boys have tried cigarettes by the age of 15. Almost every sixth adolescent is a current smoker (30-day prevalence), but only about 3% smoke daily. The higher proportion of users among both girls and boys with increasing age is particularly noticeable with alcohol, which has a higher overall prevalence than tobacco. Over 70% of 15-year-olds have drunk alcohol at least once, with every second adolescent having done so at least once in the past 30 days. Around 40% of adolescents had already been drunk by this age; about half of them had been at least once in the last month. Every sixth girl and every fifth boy has experienced cannabis by the age of 15; half of these adolescents currently use cannabis (in the past 30 days). The study found heterogeneous results for school type and family affluence. The multivariate analyses for current (30-day) substance use only identified significant differences for tobacco use by school type: adolescents who do not attend a grammar school smoke more often than those who do. Nevertheless, schoolchildren with high family affluence have a higher risk of alcohol use, and this is particularly the case with girls. However, this relationship was no longer identified after controlling for age, school type and migration background. In addition, girls and boys with a migration background have a lower risk of alcohol use or binge drinking, but girls with a one-sided migration background have an increased risk of cannabis use.

#### Comparison and interpretation of the results

The results for Germany from the 2017/18 HBSC study largely coincide with those of other studies of substance use, such as the drug affinity studies by the Federal Centre for Health Education (BZgA), the European School Survey Project on Alcohol and Other Drugs (ESPAD), the German Health Interview and Examination Survey for Children and Adolescents (KiGGS) and the international HBSC results [16, 18–22, 26, 34]. The age-specific differences in substance use reported here were also found by previous studies and can be explained by the developmental tasks and characteristics typical of adolescence, such as the adolescents’ desire to increasingly distance themselves from their parents, try out risky behaviour and overstep boundaries – especially as part of a peer group. As such, the risk of risky behaviour such as substance use increases sharply during this time [5, 35]. Other studies have also found sex-specific differences for alcohol, with higher levels of alcohol use among boys [16, 25, 36]. In the last nationwide HBSC survey, differences in tobacco use (such in regard to school type) were also observed. These results showed that girls who attended a lower secondary school (‘Hauptschule’) smoked twice as often as boys from the same type of school [24]. However, the current study only found slight sex-specific differences.

#### Results by school type, family affluence and migration background

The multivariate results presented in this article from the 2017/18 survey demonstrate school-specific differences for tobacco use only. Comparable results have also been identified by previous studies [24, 26, 37]. The association between education and tobacco use can be attributed, among other things, to the fact that tobacco prevalence is higher on non-grammar schools and that young people with a majority of friends who are smokers are more likely to smoke [38, 39]. However, no differences in tobacco use were found for family affluence. In fact, the results show that tobacco use is particularly associated with indicators of socioeconomic status that are closest to young people’s living environments (e.g. achievement at school and school type) and that these show a stronger relation to smoking behaviour than parental indicators of socioeconomic status [37]. The results also demonstrate that smoking behaviour is often highly influenced by peer group composition and school setting (school type) [40].

In contrast to the results for tobacco, higher lifetime alcohol prevalence was found among schoolchildren from socially better off families. Girls with low family affluence less often drink alcohol than those with high family affluence. However, the prevalence of current alcohol use and binge drinking are lower for grammar school pupils than for those who attend other types of school. However, the school type specific results could not be confirmed in the multivariate results. This heterogeneous relationship between family affluence, education and alcohol has also been observed internationally by several other studies [21, 41, 42]. A possible explanation could be that the first contact with alcohol often takes place in the family setting, whereas the first experiences with tobacco are largely made together with friends. Other studies indicate that alcohol is more freely available in families with higher family affluence, which means that adolescents from these families can consume alcohol more often; however, they do so more moderately, since they remain under the supervision and control of their parents [43].

The HBSC results also demonstrate that migration background plays a role in the prevalence of alcohol use. The results for both sexes show that schoolchildren with a one- or two-sided migration background try alcohol less frequently, are less likely to use it currently, and experience binge drinking less often (among boys this only applies to those with a two-sided migration background). This can be explained by cultural differences in consumption patterns, such as the fact that the liberal drinking culture in Germany treats alcohol as a common part of any celebration, a pattern that is extremely different to the picture painted in other cultures. For example, since their value system tends to advocate a culture of abstinence, the cultural and/or religiously-influenced backgrounds of adolescents from Arab countries of origin are encouraged to avoid alcohol and other drugs [44, 45].

#### Strengths and limitations

The results are based on a large representative sample of adolescents aged 11, 13 and 15 years in Germany. The HBSC study uses validated and comprehensive items to assess the use of various substances and their related social determinants. Despite these strengths, the study also has a number of limitations.

The current HBSC study did not record any information on the patterns of consumption found among the participants’ family or friends. This is important because other studies have shown that these patterns have a decisive influence, in particular, on smoking behaviour but also on alcohol use in adolescence [39, 40]. Nor did the survey ask any questions about the use of e-cigarettes or water pipes (shishas), which have recently gained in popularity and are also linked to health risks [46, 47]. The HBSC results demonstrate differences according to migration background for alcohol use, but these, among other things, could not be assigned to any particular ethnic group due to the low number of cases in specific ethic groups and for data protection reasons. As people with a migration background constitute a highly heterogeneous group, future studies could conduct a more detailed analysis of these differences. Despite these limitations, school surveys such as the HBSC remain one of the most important and robust methods of measuring substance use by adolescents and for obtaining valid and informative data in this area [48, 49].

#### Conclusions for prevention

The following conclusions for preventive measures can be derived from the results set out above: 1) since substance use increases significantly from the age of 13, prevention should be started as early as possible; 2) tobacco prevention measures should particularly be aimed at schoolchildren who do not attend grammar schools; 3) measures promoting the responsible use of alcohol should address all socioeconomic status groups but primarily adolescents from better off families. Finally, 4) cannabis prevention measures should increasingly target girls and boys with a migration background.

Although studies have identified a significant decrease in tobacco and alcohol use in Germany [18, 26, 50] and internationally [21, 51], the results indicate that substance use is still popular in adolescence. Importantly, longitudinal studies confirm that smoking behaviour remains relatively stable during the transition from adolescence to young adulthood. As such, the majority of adolescent smokers will continue to smoke in adulthood, whereas non-smokers will continue to refrain from tobacco use in adulthood [52]. Therefore, it is important that prevention begins early and aims for abstinence or at least the reduction of tobacco use. When it comes to alcohol, moderate alcohol use is often advised. However, there is also evidence that abstaining from alcohol is the healthier option, since moderate alcohol use also increases mortality [53]. It has long been discussed whether and the extent to which cannabis can be considered an entry drug into other illegal psychotropic substances and problematic patterns of consumption. Increasing numbers of studies have confirmed this trend and the fact that tobacco and alcohol use in adolescence is associated with an increased risk of problematic substance use in adulthood [54].

A large number of different factors from the adolescents’ social contexts, such as other family and school determinants (family structure, parent-child relationship, pressure at school, school environment and support at school) have proven relevant for substance use [55]. School is a particularly important setting for initiating health-promoting measures. All children and adolescents can be accessed via schools, which can either promote substance use or act as an obstacle to it. A comprehensive review of the effectiveness of school-based interventions aimed at preventing or reducing substance use concludes that school programmes that boost self-confidence and take peer resistance into account (resisting peer pressure) are more successful than prevention measures that do not. Interventions that are based on several components and include different levels (and so go beyond the individual level to include organisational changes) are more effective, especially with regard to alcohol and cannabis use [56]. The BZgA also emphasises the role of the school, alongside the family and policy level in preventing substance use. Measures that are merely aimed at providing information (such as educational programmes that provide health education) or affective elements and non-interactive measures are less effective than interactive programmes that take the social influence model into account (e.g. the peer group) as well as life skills. This applies regardless of the substance in question [57].

In summary, substance use, especially among older schoolchildren, is still widespread. Despite the decline in tobacco and alcohol use, health promotion and prevention measures should continue to apply across the board. Finally, the school setting is particularly suitable for establishing health-promoting and preventive measures, but it is essential that these measures are age-appropriate and reflect the needs of target groups [58].

## Key statements

- The results show that experiences with tobacco, alcohol and cannabis are still widespread.
- With the exception of cannabis, no significant sex-specific differences were identified for substance use; however, significant age-specific differences were found.
- Schoolchildren who do not attend a grammar school smoke more often, whereas girls with high family affluence are at greater risk of alcohol use.
- Adolescents with a migration background have a lower risk of regular alcohol use or binge drinking but face an increased risk of cannabis use (girls with one-sided migration background).
- Preventive measures should start early and take different risk groups into account depending on the substance considered.

## Figures and Tables

**Table 1:** Tobacco, alcohol and cannabis use by sex (n=2,306 girls, n=2,041 boys)* Source: 2017/18 German HBSC study

Girls			Boys		Total	
n		%	n	%	n	%
**Tobacco use (n=4,281-4,285)**						
Lifetime prevalence^1^	337	14.2	295	14.8	632	14.5
30-day prevalence^2^	170	7.0	130	6.3	300	6.7
Daily	28	1.3	24	1.4	52	1.3
**Alcohol use (n=4,261-4,267)**						
Lifetime prevalence	897	37.6	822	39.6	1,719	38.6
30-day prevalence	589	24.3	483	22.9	1,072	23.6
**Binge drinking (n=4,267-4,278)**						
Lifetime prevalence	410	17.2	352	17.1	762	17.1
30-day prevalence	182	7.4	173	8.5	355	7.9
**Cannabis use (15-year-olds only, n=1,468-1,481)**						
Lifetime prevalence	136	15.5	145	22.6	281	18.8
30-day prevalence	72	8.3	64	10.1	136	9.2

^1^ At least once in a lifetime

^2^ At least once in the last 30 days

* Percentages are based on weighted data. Absolute numbers of cases are unweighted frequencies.

**Table 2:** Tobacco, alcohol and cannabis use by sex and age (n=2,306 girls, n=2,041 boys)* Source: 2017/18 German HBSC study

	Girls			Boys		
	11 years	13 years	15 years	11 years	13 years	15 years
%		%	%	%	%	%
**Tobacco use**	**n=2,273 – 2,275**			**n=2,008 – 2,010**		
Lifetime prevalence^1^	1.1	8.2	30.4	1.3	12.5	31.3
30-day prevalence^2^	0.7	4.1	14.8	0.0	4.7	14.5
Daily	0.0	0.3	3.3	0.0	1.3	2.8
**Alcohol use**	**n=2,262 – 2,267**			**n=1,999 – 2,000**		
Lifetime prevalence	4.5	29.5	72.2	12.9	34.7	72.5
30-day prevalence	2.4	13.9	51.7	3.6	14.7	51.9
**Binge drinking**	**n=2,263 – 2,273**			**n=2,004 – 2,005**		
Lifetime prevalence	1.5	5.3	40.4	1.6	7.8	43.0
30-day prevalence	0.3	1.8	18.4	0.1	3.1	22.8
**Cannabis use (15-year-olds only)**	**n=835 – 840**			**n=633 – 641**		
Lifetime prevalence	–	–	15.5	–	–	22,6
30-day prevalence	–	–	8.3	–	–	10,1

^1^ At least once in a lifetime

^2^ At least once in the last 30 days

^*^ Percentages are based on weighted data. Absolute numbers of cases are unweighted frequencies.

**Table 3:** Logistic regression models for current substance use (30-day prevalence) by sociodemographic/socioeconomic variables for girls (tobacco and alcohol use n=2,306, binge drinking n=1,811, cannabis use n=828) Source: 2017/18 German HBSC study

	Tobacco use		Alcohol use		Binge drinking		Cannabis use	
	Model 1^*^	Model 2^**^	Model 1	Model 2	Model 1	Model 2	Model 1	Model 2
OR (95% CI)		OR (95% CI)	OR (95% CI)	OR (95% CI)	OR (95% CI)	OR (95% CI)	OR (95% CI)	OR (95% CI)
**Age group**								
11 and 13 years	Ref.	Ref.	Ref.	Ref.	Ref.	Ref.		
15 years	**7.59**	**7.63**	**11.52**	**13.44**	**21.50**	**22.43**		
	(4.81–11.99)	(4.81–12.10)	(8.79–15.10)	(10.13–17.85)	(11.31– 40.85)	(11.76–42.79)		
**School type**								
Grammar school	Ref.	Ref.	Ref.	Ref.	Ref.	Ref.	Ref.	Ref.
Other school types	**2.38**	**2.49**	0.94	1.09	1.09	1.21	1.47	1.52
	(1.49–3.79)	(1.56–3.99)	(0.72–1.22)	(0.83–1.43)	(0.73–1.63)	(0.81–1.83)	(0.84–2.57)	(0.85–2.69)
**Family affluence**								
High	Ref.	Ref.	Ref.	Ref.	Ref.	Ref.	Ref.	Ref.
Medium	0.85	0.82	0.74	0.75	0.99	1.00	0.77	0.71
	(0.53–1.36)	(0.51–1.31)	(0.54–1.02)	(0.55–1.03)	(0.61–1.61)	(0.61–1.63)	(0.43–1.38)	(0.40–1.28)
Low	0.79	0.72	**0.38**	**0.41**	0.59	0.63	0.78	0.71
	(0.44–1.40)	(0.40–1.29)	(0.25–0.58)	(0.27–0.62)	(0.32–1.10)	(0.34–1.17)	(0.38–1.62)	(0.34–1.51)
**Migration background**								
None	Ref.	Ref.	Ref.	Ref.	Ref.	Ref.	Ref.	Ref.
One-sided	0.96	0.90	**0.49**	**0.48**	**0.53**	**0.52**	**2.00**	**1.97**
	(0.55–1.67)	(0.51–1.57)	(0.33–0.72)	(0.32–0.72)	(0.28–0.98)	(0.28–0.97)	(1.04–3.84)	(1.02–3.80)
Two-sided	0.82	0.77	**0.42**	**0.44**	**0.45**	**0.46**	0.91	0.98
	(0.52–1.29)	(0.48–1.22)	(0.31–0.57)	(0.32–0.60)	(0.27–0.75)	(0.28–0.77)	(0.47–1.77)	(0.45–1.74)

OR = odds ratio, Ref. = Reference, CI = confidence interval, bold print = significant values (p <0.001)

^*^ Model 1 = age-adjusted (except for cannabis use as only data from 15-year-olds were included)

^**^ Model 2 = adjusted for age, school type, family affluence and migration background

**Table 4:** Logistic regression models of current substance use (30-day prevalence) by sociodemographic/socioeconomic variables for boys (tobacco and alcohol use n=2,041, binge drinking n=1,618, cannabis use n=623) Source: 2017/18 German HBSC study

	Tobacco use		Alcohol use		Binge drinking		Cannabis use	
	Model 1^*^	Model 2^**^	Model 1	Model 2	Model 1	Model 2	Model 1	Model 2
OR (95% CI)		OR (95% CI)	OR (95% CI)	OR (95% CI)	OR (95% CI)	OR (95% CI)	OR (95% CI)	OR (95% CI)
**Age group**								
11 and 13 years	Ref.	Ref.	Ref.	Ref.	Ref.	Ref.		
15 years	**8.78**	**8.82**	**11.41**	**12.33**	**22.38**	**22.38**		
	(5.47–14.09)	(5.48–14.19)	(8.72–14.94)	(9.34–16.28)	(12.96–38.67)	(12.94–38.71)		
**School type**								
Grammar school	Ref.	Ref.	Ref.	Ref.	Ref.	Ref.	Ref.	Ref.
Other school types	**1.94**	**1.91**	0.98	1.10	1.15	1.18	1.34	1.36
	(1.20–3.12)	(1.18–3.09)	(0.75–1.29)	(0.83–1.46)	(0.77–1.71)	(0.79–1.77)	(0.77–2.31)	(0.78–2.37)
**Family affluence**								
High	Ref.	Ref.	Ref.	Ref.	Ref.	Ref.	Ref.	Ref.
Medium	1.08	1.01	1.02	1.06	1.31	1.32	0.90	0.84
	(0.61–1.92)	(0.56–1.80)	(0.72–1.43)	(0.75–1.51)	(0.78–2.19)	(0.79–2.22)	(0.50–1.61)	(0.47–1.51)
Low	1.55	1.42	**0.66**	0.81	0.99	1.08	0.80 (	0.68
	(0.84–2.87)	(0.76–2.67)	(0.44–0.99)	(0.54–1.24)	(0.55–1.80)	(0.59–1.98)	0.38–1.67)	(0.32–1.45)
**Migration background**								
None	Ref.	Ref.	Ref.	Ref.	Ref.	Ref.	Ref.	Ref.
One-sided	1.08	1.06	**0.51**	**0.52**	0.60	0.59	1.39	1.44
	(0.56–2.08)	(0.55–2.05)	(0.33–0.80)	(0.33–0.82)	(0.30–1.16)	(0.30–1.16)	(0.63–3.08)	(0.65–3.20)
Two-sided	0.95	0.83	**0.31**	**0.32**	**0.60**	**0.60**	1.42	1.47
	(0.59–1.52)	(0.51–1.35)	(0.22–0.44)	(0.23–0.45)	(0.38–0.94)	(0.37–0.95)	(0.79–2.57)	(0.80–2.71)

OR = odds ratio, Ref. = Reference, CI = confidence interval, bold print = significant values (p <0.001)

^*^ Model 1 = age-adjusted (except for cannabis use as only data from 15-year-olds were included)

^**^ Model 2 = adjusted for age, school type, family affluence and migration background
